# Drug information resources used by nurse practitioners and collaborating physicians at the point of care in Nova Scotia, Canada: a survey and review of the literature

**DOI:** 10.1186/1472-6955-5-5

**Published:** 2006-07-06

**Authors:** Andrea L Murphy, Mark Fleming, Ruth Martin-Misener, Ingrid S Sketris, Mary MacCara, David Gass

**Affiliations:** 1 Dalhousie University, School of Nursing, Halifax, Nova Scotia, Canada; 2 Dalhousie University, College of Pharmacy, Halifax, Nova Scotia, Canada; 3 IWK Health Centre, Halifax, Nova Scotia, Canada; 4 St. Mary's University, Psychology Department, Halifax, Nova Scotia, Canada; 5 Department of Family Medicine, Dalhousie University, Halifax, Nova Scotia, Canada; 6 At the time of research, Primary Health Care Section, Nova Scotia Department of Health, Halifax, Nova Scotia, Canada

## Abstract

**Background:**

Keeping current with drug therapy information is challenging for health care practitioners. Technologies are often implemented to facilitate access to current and credible drug information sources. In the Canadian province of Nova Scotia, legislation was passed in 2002 to allow nurse practitioners (NPs) to practice collaboratively with physician partners. The purpose of this study was to determine the current utilization patterns of information technologies by these groups of practitioners.

**Methods:**

Nurse practitioners and their collaborating physician partners in Nova Scotia were sent a survey in February 2005 to determine the frequency of use, usefulness, accessibility, credibility, and current/timeliness of personal digital assistant (PDA), computer, and print drug information resources. Two surveys were developed (one for PDA users and one for computer users) and revised based on a literature search, stakeholder consultation, and pilot-testing results. A second distribution to nonresponders occurred two weeks following the first. Data were entered and analysed with SPSS.

**Results:**

Twenty-seven (14 NPs and 13 physicians) of 36 (75%) recipients responded. 22% (6) returned personal digital assistant (PDA) surveys. Respondents reported print, health professionals, and online/electronic resources as the most to least preferred means to access drug information, respectively. 37% and 35% of respondents reported using "both print and electronic but print more than electronic" and "print only", respectively, to search monograph-related drug information queries whereas 4% reported using "PDA only". Analysis of respondent ratings for all resources in the categories print,  health professionals and other, and online/electronic resources, indicated  that the Compendium of Pharmaceuticals and Specialties and pharmacists  ranked highly for frequency of use, usefulness, accessibility, credibility,  and current/timeliness by both groups of practitioners. Respondents' preferences and resource ratings were consistent with self-reported methods for conducting drug information queries. Few differences existed between NP and physician rankings of resources.

**Conclusion:**

The use of computers and PDAs remains limited, which is also consistent with preferred and frequent use of print resources. Education for these practitioners regarding available electronic drug information resources may facilitate future computer and PDA use. Further research is needed to determine methods to increase computer and PDA use and whether these technologies affect prescribing and patient outcomes.

## Background

### Challenges with knowledge management for health care professionals

In 1986, Haynes et al. published a series of 6 articles entitled "how to keep up with the medical literature" in an effort to help clinicians with information management, but this challenge has not decreased in last two decades [1-6]. Alper et al. suggest that maintaining currency with relevant literature in primary care would "require 627.5 hours per month, or about 29 hours per weekday, or 3.6 full-time equivalents of physician effort" [7]. The volume of information associated with keeping up to date is frequently cited as a barrier [8]. It is estimated that annually there are approximately 10,000 new randomized trials in MEDLINE and over 450,000 clinical trials identified by the Cochrane Collaboration [9,10]. Keeping up to date has been described with several analogies including clinicians attempting to drink water from a fire hose and swimming in rivers of clinical research with unprecedented depth, velocity, and turbulence [11,12].

Difficulties with dissemination of research evidence and keeping up to date on pharmacotherapeutic interventions are reported despite the development of tools such as clinical practice guidelines and systematic reviews that are intended to reduce the need for practitioners to evaluate original research [13]. To complicate matters further, there are often issues of credibility, timeliness, and volume of clinical practice guidelines and reviews. Many guidelines are criticized for their methodological development. Shaneyfelt et al. reviewed 279 guidelines for methodological standards from peer reviewed medical literature [14]. These authors found that only 51%, 33.6%, and 46% adhered to standards on guideline development and format, evidence identification and summary, and formulation of recommendations, respectively [14]. A Canadian review on drug therapy guidelines found significant variation in quality depending on the developer [13]. Approximately 25% of guidelines were not recommended for use in practice by the appraisers' criteria [13]. As an example of the volume of clinical practice guidelines available, eleven recent guidelines on community acquired pneumonia exist [15]. To add to the complexities involved with keeping current with pharmacotherapeutic management strategies, as of 2000, there were over 22,000 drug products approved for sale in Canada for human use [16].

There is also considerable debate regarding what constitutes "evidence" in practice, which contributes to confusion for clinicians [17,18]. Sim et al. succinctly describe the gap between evidence and action as difficulties with obtaining, systematically reviewing, applying in context, and measuring the outcome following application of evidence [19].

### Maintaining competence – nurse practitioners as a new group of prescribers

Competencies for nurse practitioners (NPs) on a local and international level include critically appraising and applying literature and research findings in practice [20-23]. The Canadian Nurses Association (CNA) has developed the Canadian Nurse Practitioner Core Competency Framework that describes the knowledge, skills, judgment, and attributes required for practice. Evidence based practice is integral to pharmacotherapeutic interventions and prescribing competencies [23]. The National Prescribing Centre, an organization of the National Health Service in the UK, describes several competencies around information needs relevant to prescribing and emphasis is placed on using relevant and up to date information in various formats (e.g. print, electronic, verbal). Several related competencies include understanding advantages and disadvantages of information sources and the currency of resources [21]. Researchers in the US developed NP informatics competencies for integration into advanced nursing practice curricula [24]. Competencies related to informatics knowledge include critical analysis of data and information for use in evidence based practice, evaluating and applying relevant information, synthesizing best evidence, and using optimal search strategies to locate clinically sound and useful studies from information resources [24]. Achieving and maintaining competence in these domains as well as a solid foundation in pharmacology is necessary to support NPs in their relatively new role as a prescriber [25-27].

### Knowledge management and information seeking behaviours among nurse practitioners and physicians

Information seeking behaviours of physicians are better documented than NPs [11]. Information related to diagnosis is important to both groups but drug therapy queries may occur more frequently with NPs [28-33]. Research on nurses' behaviours related to information seeking is available from the hospital setting [33-35] but the generalizability of these behaviours to NPs with a prescribing role is unclear. Differences in nursing roles, responsibilities, and legislation, including prescriptive authority, exist depending on the country of practice.

### Nurse practitioners and their collaborating physician partners in Nova Scotia

Nova Scotia is a Canadian province with a population of approximately 942,000 [36]. The province is divided into six health zones that include nine district health authorities, one of which includes the provincial capital and is considered to be urban [37,38]. Health care service delivery is challenging due to many factors including the rural nature of the province, which is estimated to be 60% of population [37,39].

Starting in 1998, the Nova Scotia Department of Health led an initiative to explore different methods of delivering, managing, and funding primary care services. The Strengthening Primary Care in Nova Scotia Communities Initiative (SPCI) was established with the selection of four primary care demonstration sites where a primary health care NP was hired to practice collaboratively with one or more family/general physicians and other members of an interdisciplinary team. Each demonstration site adopted alternative (non fee-for-service) physician payment mechanisms and used electronic patient records (EPRs) to support service delivery [41]. Demonstration sites participated in project evaluation components that included, but were not limited to, NP roles, alternative fee structures, consumer satisfaction, and implementation and integration of EPRs [41,42].

Legislation to allow NPs to practice collaboratively with physicians in Nova Scotia was passed in 2002, part way through the SPCI project [39]. Prescriptive authority granted through legislation authorizes NPs to prescribe from a schedule of drugs [43,44]. At the time of conducting this research project, 16 primary health care NPs were in active practice [43].

The EPR component of the SPCI project evaluation provided information on the use of technologies in the community context. Results from the implementation process indicated that considerable attention is required for technology literacy, time for training, and selection of software for EPRs [41]. Although the majority of community-based, non-institutional clinical practice settings in Nova Scotia primarily operate with paper-based charting systems, there is a movement toward integrating electronic technologies, including the EPR, in practice among health care providers, administrators, and the provincial government. In addition to recording patient visit information, a component of the EPR package serves to provide drug information resources.

Drug therapy information resources for NPs and nurse prescribers have frequently been described as essential in supporting practice [25,28,29]. The role of NPs is relatively new in Canada [39] and there is limited information available to indicate the type of resources (e.g. print, electronic, EPR based) these prescribers use for drug and therapeutic information queries at the point of care. It is unknown as to whether differences exist regarding types of resources used, drug information needs, and utilization patterns among NPs and collaborating physician partners. Some research has suggested that the degree of multidisciplinary team functioning relates to the adoption of technology or innovations in practice but more research is required to determine the extent of these relationships [45,46].

The use of EPR technology is increasing in Nova Scotia but little information is available regarding the readiness of practitioners for use of specific features such as drug information resources. Based on the EPR related results of the SPCI evaluation, use of these functions could be challenging without proper facilitation. The purpose of the survey for this research was to describe drug information resources used by NPs and their collaborating physician partners at the point of care. The results of the survey will be used to guide further technology implementation strategies and stimulate further discussion around drug information resource usage at the point of care.

## Methods

### Survey development

Survey development involved three stages including identification of important content areas, development of draft questions, and survey refinement.

Identifying important content areas for inclusion in the survey involved conducting a comprehensive English language literature search, consultation with relevant stakeholders (e.g. members of the Nova Scotia Department of Health), and input from subject matter experts at Dalhousie University. The literature review was conducted using the following bibliographic databases: PubMed, Cumulative Index to Nursing and Allied Health Literature (CINAHL), International Pharmaceutical Abstracts (IPA), and Web of Science Citation Databases. Hand and electronic searching of relevant journals was also conducted. Broad search terms were used without limits on publication date or place as nurse practitioner titles, roles and scopes of practice, and terminology regarding technology vary nationally and internationally. Some examples of terms used included nurse practitioner, nurse prescriber, nurse clinicians, district nurse, health visitor, drug information resources, drug information services, information needs, and information technology.

The draft survey was reviewed by the research team to reduce the number of items and improve clarity. The layout of the questionnaire was carefully examined to ensure that it was easy to follow and complete. Research results from a previous investigation of Nova Scotian physicians' behaviours regarding drug information were also used to further revise the survey [47]. This draft questionnaire was pilot tested by two out of province NPs and one physician. The results of the pilot were used to make final revisions to the survey. Based on pilot-testing feedback and investigator consensus, the final survey was divided into 2 versions, one for personal digital assistant (PDA) users and one for computer users.

The 10 page surveys for PDA and computer users had 5 or 6 sections, respectively, and 37 questions, many with multiple parts. The survey content included demographics, computer or PDA use and experience, drug and therapeutic resource use and preferences, PDA future use, perceived barriers and facilitators to PDA use, and technology training preferences.

Section one contained demographic questions such as gender, age, job title, volume of patients, and EPR availability in the practice setting. Section two was designed to determine PDA or computer use and experience in the practice setting with questions regarding length of use, costs, and work versus home usage. This section also addressed usage and rating of different drug information resources. Resource ratings were based on the frequency of usage, usefulness, accessibility, credibility, and current/timeliness. Resources were grouped as print (i.e. books, journals, and clinical practice guidelines), online/electronic resources, and health professionals and other. Respondents used 5-point Likert scales (strongly agree to strongly disagree) for rating opinions related to resources. A rating of 6 (not applicable, I do not use this resource) was also included for respondents who did not use a particular resource. Frequency of searching for specific information was rated on a 3-point Likert scale (frequently to never). The final sections of the survey included categorical, open-ended, and Likert scale questions regarding preferred resources, technology barriers, PDA future use, and technology training preferences. Copies of the surveys are attached as an appendix in PDF format [see additional file 1 and 2] or can also be accessed from the Initiative for Medication Management, Policy Analysis, Research & Training (IMPART) website [48].

Ethics approval for the survey was granted through Dalhousie University Research Ethics Board on February 3, 2005.

### Survey population

Licensed, actively practicing, primary health care NPs (n = 16) and their collaborating physician partners (n = 21) were eligible to participate.

### Survey procedures

The survey recruitment procedures were based on the methods of Dillman [49] and Salant and Dillman [50]. Survey packages contained a cover letter, separate surveys for PDA and computer users, and a return self-addressed stamped envelope. The covering letter instructed respondents to self-select the appropriate survey (either PDA or computer) based on their drug information seeking behaviours. Participants who had used a PDA at any time were instructed to complete the PDA version of the survey. Those who had never used a PDA for drug information were instructed to complete the computer version of the survey. Several strategies were used to optimize response rate and included: personalized cover letters, coloured paper for surveys, stamped return envelopes, follow-up mailing, and a priority post mailing [51]. The covering letter included coloured logos of Dalhousie University and the Nova Scotia Department of Health representing the investigator affiliations and endorsement of the project.

A master mailing list with names and addresses of NPs and their collaborating physician partners was created. To maintain confidentiality of respondents, a number placed on the bottom right corner of each survey corresponded to a name on the confidential master mailing sheet. The postage paid return envelopes were addressed to the research coordinator at the School of Nursing, Dalhousie University, who matched respondents to the mailing list from the first distribution. The cross-referenced mailing list was not accessible to those entering or analysing data. The research coordinator sent the second distribution to those who had not initially responded. A fluorescent coloured page was included in the second mailing to notify recipients of the second and final mailing status. The second mailing followed 2 weeks after the initial mailing (February 2005). The surveys were sent via Xpresspost™ through Canada Post.

### Data analyses

#### Quantitative

Data were entered and analysed in Statistical Package for Social Sciences (SPSS) (version 11.5 for Windows). Five surveys were randomly selected as a check for accuracy of data entry. Descriptive statistics were used to describe resource usage by practitioners. Chi Square (Fisher's Exact when cell count less than 5) analyses were used to determine differences in computer or PDA use based on predetermined variables (e.g. high speed Internet connection, number of patients per day). Mann Whitney U tests were used to compare physician and NPs Likert scale ratings (1 = strongly agree to 5 = strongly disagree) of resource use. Physician and NP rankings of all resources (print, online/electronic, and health professionals and other) were determined from means of Likert scale ratings (1 = strongly agree, 5 = strongly disagree) for each of the pre-specified characteristics (e.g. frequency of use, accessibility, etc.) and the frequency of use of the resources. The best rankings were assigned for the lowest mean scores and the largest number of the sample using a resource. These rankings (ranks based on mean and ranks based on sample) were then entered into a formula to calculate an overall rank. The formula includes: rank = [(rank according to % of sample using the resource + rank based on mean score) ÷ 2]. This formula was used to account for mean scores based on small samples as these numbers could potentially over or underestimate the value of a resource. Ratings of 6 (i.e. not applicable, I do not use this resource) were excluded from the analyses.

#### Qualitative

Comments were entered in a word-processing program and organized by type of respondent (PDA versus computer) and question number. The coded survey number and respondent type (NP or physician) were also included next to comments. Investigators determined themes and categorized comments based on previous experience, knowledge, and familiarity with the topic.

## Results

Surveys were completed and returned by 75% of eligible participants (27 of 36). One physician survey was undeliverable. The response rates from within the NP and physician samples were 88% and 65%, respectively. Complete demographic information is available in Table 1.

**Table 1:** Demographics of nurse practitioner and collaborating physician partner respondents.

Characteristic	Descriptor	N	(%)
Practitioner type	Nurse practitioner	14*	52
	Physician	13	48
Gender	Male	9	33
	Female	18	67
Age	≤ 25	2	7
	26 – 35	4	15
	36 – 55	16	59
	56 – 65	4	15
	≥ 66	1	4
Survey type	Personal digital assistant^†^	6	22
	Computer	21	78
Patient volume	Mean patients/day/week^‡^		
	<15	11	41
	16–25	11	41
	26–35	2	7
	36–45	2	7
	>46	1	4
Technologies available	High speed internet at work^§^		
	Yes	15	56
	No	10	37
	Electronic Patient Record		
	Yes	10	37
	No	17	63

* represents 88% (14/16) response rate within the nurse practitioner population.

† 1 and 5 personal digital assistant surveys received from nurse practitioners and general practitioners, respectively.

‡ mean number of patients seen by the practitioner per day in a week.

§totals do not add up to 100% due to 2 non-responses.

### Methods for accessing resources and self-reported resource use

Resource use was similar amongst practitioners. Respondents indicated that print resources (mean 4.56, SD 0.80), health professionals (mean 3.26, SD 0.90), and online/electronic resources (mean 2.70, SD 1.20) were the preferred method (1 = least preferred to 5 = most preferred) for accessing drug information. Thirty-seven percent of respondents reported that searching for specific questions related to drug information (e.g. usual dosage, duration of therapy) was conducted using both print and electronic resources (but print use greater than electronic) (Table 2). The preferred means (i.e. print) to access resources was consistent with the most common means of conducting searches for specific drug information queries.

**Table 2:** Most common means to search for specific drug information queries by nurse practitioners and collaborating physicians

**Method to conduct searching**	**% respondents**
Print use is greater than electronic, although both used	37
Print only	35
Electronic use is greater than print, although both used	8
Print used more than electronic and/or personal digital assistant	4
Print and electronic used equally	4
Print, electronic, and personal digital assistant used equally	4
Personal digital assistant used more than electronic and/or print	4
Personal digital assistant only	4

Respondents' ratings for pre-specified print, online/electronic, and professional resources and other, based on means from Likert scales and number of respondents using the resources, are presented in Tables 3, 4, and 5. Of all resources within the print, online/electronic, and health professionals or other categories, NPs and physicians rated the Compendium of Pharmaceuticals and Specialties (CPS) [52] and pharmacists as the top two most frequently used resources for providing drug and therapeutic information. Physicians rated other physicians as the third most frequently used resource. The book Therapeutic Choices [53] ranked third for NPs. Based on written feedback, physicians and NPs consulted pharmacists and other physicians most frequently. The CPS and pharmacists were also ranked as the top two resources overall in terms of usefulness, accessibility, credibility, and current/timeliness for physicians. Rankings by NPs were similar for usefulness, accessibility, and credibility. NPs ranked pharmacists, Therapeutic Choices, and academic detailing first and the CPS as second for current/timeliness.

**Table 3:** Print resource ratings (1 = strongly agree, 5 = strongly disagree) by physicians and nurse practitioners

	**Print**																			
	
	**CPS**				**TC**				**Specialty books**				**Print journals**				**Print CPGs**			
	
	**Physician** **(N = 13)**		**NP** **(N = 14)**		**Physician** **(N = 8)**		**NP** **(N = 13)**		**Physician** **(N = 10)**		**NP** **(N = 12)**		**Physician** **(N = 10)**		**NP** **(N = 12)**		**Physician** **(N = 12)**		**NP** **(N = 14)**	
	Mean (SD)	Rank*	Mean (SD)	Rank*	Mean (SD)	Rank*	Mean (SD)	Rank*	Mean (SD)	Rank*	Mean (SD)	Rank*	Mean (SD)	Rank*	Mean (SD)	Rank*	Mean (SD)	Rank*	Mean (SD)	Rank*
Used frequently	1.62 (0.96)	1	1.29 (0.61)	1	2.75 (1.39)	8.5	1.54 (0.78)	2.5	2.30 (0.67)	4	1.83 (0.83)	3.5	2.70 (1.25)	7	2.25 (0.87)	5	2.58 (1.00)	5.5	2.14 (0.86)	3.5
Useful	1.46 (0.52)	1	1.50 (0.65)	1.5	2.00 (1.00)	6	1.69 (1.11)	2.5	1.88 (0.83)	4	1.75 (0.87)	3.5	2.40 (0.97)	7.5	1.92 (0.67)	5.5	2.25 (0.87)	5.5	2.00 (0.68)	5
Accessible	1.31 (0.48)	1	1.36 (0.63)	1	2.14 (1.21)	5	1.38 (0.51)	2	2.13 (0.83)	3.5	1.75 (0.75)	3.5	2.70 (1.06)	8	2.33 (0.99)	5.5	2.83 (0.94)	7.5	2.36 (0.84)	5
Credible	1.38 (0.65)	1	1.36 (0.50)	1	1.71 (0.95)	4.5	1.38 (0.65)	2	2.00 (0.87)	5.5	1.67 (0.78)	3.5	2.30 (1.06)	7.5	1.83 (0.83)	4.5	2.17 (0.72)	5.5	2.00 (0.88)	4.5
Current/timely	1.85 (0.69)	2.5	1.86 (0.77)	3	2.14 (1.21)	6.5	1.54 (0.66)	2	2.25 (1.03)	6.5	1.83 (0.83)	3.5	2.10 (0.88)	5	1.92 (0.79)	4.5	2.58 (0.90)	6.5	2.21 (0.89)	6.5

Abbreviations: NP: Nurse practitioner; CPS: Compendium of Pharmaceuticals and Specialities; TC: Therapeutic Choices

CPGs: Clinical practice guidelines

* Rank: calculated based on the formula: [(rank according to % of sample using the resource + rank based on mean score) ÷ 2]. Ranks based on the % of the sample using the reference and individual ranks for mean scores were assigned within the groups used frequently, usefulness, accessibility, credibility, and current/timeliness for all resources regardless of their category (i.e. print, online/electronic, health professionals or other).

**Table 4:** Online/electronic resource ratings (1 = strongly agree, 5 = strongly disagree) by physicians and nurse practitioners

	**Online/electronic**																			
	
	**Online journals**				**Online bibliographies**				**Electronic CPGs**				**Cochrane**				**Specialty websites**			
	
	**Physician** **(N = 5)**		**NP** **(N = 9)**		**Physician** **(N = 4)**		**NP** **(N = 7)**		**Physician** **(N = 7)**		**NP** **(N = 11)**		**Physician** **(N = 6)**		**NP** **(N = 7)**		**Physician** **(N = 2)**		**NP** **(N = 7)**	
	Mean (SD)	Rank*	Mean (SD)	Rank*	Mean (SD)	Rank*	Mean (SD)	Rank*	Mean (SD)	Rank*	Mean (SD)	Rank*	Mean (SD)	Rank*	Mean (SD)	Rank*	Mean (SD)	Rank*	Mean (SD)	Rank*
Used frequently	2.40 (1.34)	7.5	2.44 (1.42)	7	3.00 (0.82)	11	2.86 (1.07)	9.5	2.29 (1.11)	5	2.45 (1.04)	7	3.00 (1.09)	10	3.43 (1.13)	11	3.00 (0.00)	12	1.86 (0.69)	5.5
Useful	2.20 (1.30)	8.5	2.29 (1.50)	8	2.40 (0.55)	10.5	2.43 (1.13)	9.5	2.14 (0.90)	7	2.18 (0.87)	7	2.33 (0.82)	9	2.00 (0.82)	7.5	3.00 (0.00)	13	1.86 (0.69)	6.5
Accessible	2.60 (1.14)	9.5	2.38 (1.60)	7.5	2.40 (0.55)	8	2.86 (0.69)	9.5	2.14 (0.90)	5.5	2.36 (0.92)	6.5	2.50 (1.05)	8	2.43 (0.53)	8.5	3.00 (0.00)	13.5	1.86 (0.69)	5.5
Credible	2.20 (1.30)	9.5	2.29 (1.50)	7.5	1.80 (0.84)	7	2.43 (0.98)	9	2.00 (0.82)	7	2.09 (0.94)	6.5	1.67 (0.52)	5	1.86 (0.90)	6.5	3.00 (0.00)	13	1.86 (0.69)	6.5
Current/timely	2.20 (1.30)	8.5	2.14 (1.57)	7	1.60 (0.89)	5.5	2.43 (0.98)	10	2.00 (0.82)	6	2.18 (1.08)	7	1.83 (0.75)	5.5	2.00 (0.89)	6.5	3.00 (0.00)	12.5	2.00 (0.82)	6.5

Abbreviations: NP: Nurse practitioner; CPGs: Clinical practice guidelines

* Rank: calculated based on the formula: [(rank according to % of sample using the resource + rank based on mean score) ÷ 2]. Ranks based on the % of the sample using the reference and individual ranks for mean scores were assigned within the groups used frequently, usefulness, accessibility, credibility, and current/timeliness for all resources regardless of their category (i.e. print, online/electronic, health professionals or other).

**Table 5:** Health professionals and other resource ratings (1 = strongly agree, 5 = strongly disagree) by physicians and nurse practitioners

	**Health professionals and other**																			
	
	**Physicians**				**Nurses**				**Pharmacists**				**Other health professionals (e.g. dietician)**							
	
	**Physician** **(N = 13)**		**NP** **(N = 14)**		**Physician** **(N = 12)**		**NP** **(N = 13)**		**Physician** **(N = 13)**		**NP** **(N = 14)**		**Physician** **(N = 9)**		**NP** **(N = 12)**					
	Mean (SD)	Rank*	Mean (SD)	Rank*	Mean (SD)	Rank*	Mean (SD)	Rank*	Mean (SD)	Rank*	Mean (SD)	Rank*	Mean (SD)	Rank*	Mean (SD)	Rank*				
Used frequently	2.31 (1.11)	3	1.86 (0.95)	3	2.75 (0.87)	6.5	2.62 (0.96)	7	2.00 (1.29)	1.5	1.43 (0.65)	1.5	2.56 (0.88)	6.5	2.25 (0.62)	5				
Useful	1.85 (0.80)	2	1.50 (0.65)	1.5	2.33 (0.78)	6	1.85 (0.55)	4	1.77 (0.83)	1.5	1.43 (0.65)	1	2.44 (0.53)	8.5	1.83 (0.72)	4				
Accessible	2.46 (0.88)	4	2.00 (1.04)	3.5	2.50 (0.80)	5	2.46 (0.78)	7	1.69 (0.63)	1.5	1.50 (0.65)	2	2.67 (0.50)	8	2.00 (0.43)	4.5				
Credible	2.08 (0.76)	4.5	1.64 (0.75)	2	2.58 (0.67)	7.5	1.85 (0.55)	4.5	1.85 (0.90)	3.5	1.36 (0.50)	1	2.44 (0.53)	8.5	1.83 (0.58)	4.5				
Current/ timely	2.25 (0.87)	5	2.00 (0.68)	4	2.58 (0.67)	6.5	2.08 (0.49)	5	1.77 (0.73)	1.5	1.64 (0.63)	2	2.56 (0.53)	7.5	1.92 (0.51)	4.5				

	**Online chat/discussion groups**				**Academic detailing**				**Regional DI service**				**Pharmaceutical company DI**				**Pharmaceutical representatives**			
	
	**Physician** **(N = 2)**		**NP** **(N = 5)**		**Physician** **(N = 12)**		**NP** **(N = 12)**		**Physician** **(N = 3)**		**NP** **(N = 7)**		**Physician** **(N = 2)**		**NP** **(N = 6)**		**Physician** **(N = 11)**		**NP** **(N = 12)**	
	Mean (SD)	Rank*	Mean (SD)	Rank*	Mean (SD)	Rank*	Mean (SD)	Rank*	Mean (SD)	Rank*	Mean (SD)	Rank*	Mean (SD)	Rank*	Mean (SD)	Rank*	Mean (SD)	Rank*	Mean (SD)	Rank*

Used frequently	3.50 (0.71)	13.5	2.60 (1.14)	9.5	2.50 (0.91)	4.5	2.42 (1.00)	5.5	3.33 (0.58)	12.5	3.29 (0.76)	10.5	3.50 (0.71)	13.5	3.67 (1.03)	12	3.18 (1.25)	8	3.17 (1.11)	8.5
Useful	2.50 (0.71)	12.5	2.40 (0.89)	10	1.92 (0.67)	3.5	1.85 (1.14)	4.5	2.00 (1.00)	8.5	2.86 (0.90)	10.5	3.00 (0.00)	13	3.50 (1.05)	11.5	3.18 (1.08)	9	2.58 (0.79)	8.5
Accessible	3.00 (0.00)	13.5	2.20 (0.84)	7.5	2.58 (1.00)	5.5	2.38 (1.33)	6.5	2.33 (0.58)	8	3.14 (0.69)	10	3.00 (0.00)	13.5	3.33 (1.21)	11	2.91 (1.37)	8.5	3.08 (1.24)	8
Credible	3.00 (0.00)	13	2.40 (0.89)	9.5	1.83 (0.72)	3.5	1.77 (0.93)	4	2.00 (1.00)	9	2.86 (0.90)	9.5	3.00 (0.00)	13	3.67 (0.82)	11	3.27 (1.01)	9	2.92 (0.90)	8.5
Current and timely	3.00 (0.00)	12.5	2.20 (0.84)	9.5	2.00 (0.95)	3.5	1.46 (0.52)	2	2.00 (1.00)	8	2.86 (0.90)	10.5	3.00 (0.00)	12.5	3.50 (1.05)	11.5	2.91 (1.22)	7.5	2.42 (0.79)	8

Abbreviations: NP: Nurse practitioner; DI: Drug information. * Rank: calculated based on the formula: [(rank according to % of sample using the resource + rank based on mean score) ÷ 2]. Ranks based on the % of the sample using the reference and individual ranks for mean scores were assigned within the groups used frequently, usefulness, accessibility, credibility, and current/timeliness for all resources regardless of their category (i.e. print, online/electronic, health professionals or other).

Within the online/electronic category, electronic clinical practice guidelines (eCPGs) were rated the highest for all characteristics (e.g. usefulness, credibility). Although eCPGs were highly ranked, approximately 30% of the sample reported not using this resource. Other resources in this category were infrequently used based on respondents' self-reports.

Pharmaceutical industry representatives were used as a source of drug information by 85% and 86% of physicians and NPs, respectively (Table 5). This was higher than regional drug information services (used by 23% of physicians and 50% of NPs). After exclusion of traditional health professionals (i.e. physicians, nurses, pharmacists, allied health) in the health professionals and other category, pharmaceutical industry representatives received rankings for second or third for frequency of use, usefulness, accessibility, credibility, and current/timeliness, based on means and number of respondents using this resource (data not shown).

### Differences between nurse practitioners and physicians

A series of Mann Whitney U tests were used to compare the responses of NPs and physicians on their use of print, online/electronic, and health professional resources. In total 95 statistical tests were conducted. The large number of tests increases the likelihood of a type I error as five significant differences would be expected by chance alone at an alpha threshold of 0.05. It is therefore important to treat these results with caution. A limited number of statistically significant (p < 0.05) differences were identified between physicians and NPs and are reported in Table 6. Therapeutic Choices differed significantly for frequency of use with more NPs making use of this resource. Allied health professionals significantly differed between NPs and physicians for accessibility and current/timeliness while NPs were more in agreement with these characteristics of the resource. Nurse colleague credibility and current/timeliness was rated significantly higher by NPs versus physicians.

**Table 6:** Significant Mann Whitney U results (p < 0.05) of nurse practitioner versus physician ratings for resources.

		Resources and characteristic				
Test statistic and p value		Therapeutic Choices is used frequently	Nurses are credible	Nurse are current/timely	Allied health professionals are accessible	Allied health professionals are current/timely
Mean score	Physicians	15.27	16.83	16.04	14.67	14.28
	NP	12.82	9.46	10.19	8.25	8.54
Mann-Whitney U		24.50	32.00	41.50	21.00	24.50
p value		p = 0.045	p = 0.011	p = 0.046	p = 0.018	p = 0.034

Abbreviations: NP: Nurse practitioner

### Factors influencing electronic technology use at the point of care

Factors such as gender, age, practitioner type (NP vs physician), accessibility, technical support, Internet connection speed, patient volume, presence of an EPR, and home computer use were examined to determine if they were associated with the use of a work computer to search for drug information at the point of care. No statistically significant associations were found (Fisher's Exact).

### Additional resources from respondent comments

Respondents indicated other resources and programs, such as clinical calculators, that they would like to access from their computer or PDA. The top three resources that were desired included Canadian clinical practice guidelines, patient education information, and ability to track clinical activities/statistics. Further comments from two NP computer survey respondents revealed that a resource on drug interactions and dosages would be desired. One other NP also indicated "up to date info [sic] on drugs to treat various illnesses ie doseage [sic], length of use etc."

### Computer or personal digital assistant use in practice

Approximately 50% of computer survey respondents reported using their work computers for searching drug or therapeutic information related to patient care. Of those respondents, just over half (54%) also reported using their home computer for this purpose. Sixty-seven and 17% of PDA survey respondents reported using their PDA for searching drug or therapeutic information related to patient care at work and home, respectively.

### Searching on a weekly basis for specific information related to drugs

Of the 24 specified categories of drug information included in the survey, the majority were reported as infrequently searched and a smaller percentage as never searched by respondents (data not shown). The top three categories rated as frequently searched were side effects, adult or usual drug dosage, and most appropriate drug for an indication. (Table 7)

**Table 7:** Mean frequency of specific drug information queries searched on a weekly basis (frequently = 1, infrequently = 2, never = 3).

**Category**	**Frequency mean**	**SD**
Side effects	1.56	0.75
Adult or usual drug dosage	1.67	0.68
Most appropriate drug for indication	1.78	0.85
Drug interactions	1.81	0.79
Geriatric drug dosage	1.85	0.72
Information on new drugs	1.89	0.80
Pediatric drug dosage	1.96	0.71
Dosage forms (e.g. liquid)	1.96	0.76
Length of therapy	1.96	0.81
Indications	2.00	0.78
Dosage adjustment in organ dysfunction (e.g. renal)	2.07	0.73
Drug use in pregnancy and/or lactation	2.11	0.75
Pharmacokinetics (e.g. half-life, metabolism, excretion)	2.11	0.80
Non-prescription/Over the counter drug information	2.15	0.60
Herbal therapy information	2.19	0.62
Mechanism of action	2.19	0.68
Identification of drugs	2.27	0.67
Cost of drugs	2.27	0.83
New indication(s) for older drugs	2.33	0.62
Monitoring (e.g. phenytoin levels, bloodwork frequency)	2.33	0.68
Toxicology/treatment of overdose or poisoning	2.37	0.63
Formulary status (e.g. Nova Scotia Formulary)	2.41	0.69
Criteria for formulary exceptions status	2.42	0.76
Non-medicinal content of drugs (e.g. dyes)	2.56	0.51

### Issues related to personal digital assistants

Respondents reported their level of agreement with statements related to how PDAs may influence their practice. The statements included aspects of workload (organization and paper work), convenience, and improving quality of care and patient outcomes. (Table 8) Respondents agreed that PDAs are a convenient resource but indicated that PDAs would not decrease paperwork or improve patient health outcomes.

**Table 8:** Respondents' agreement (Likert scale of 1 = strongly agree to 5 = strongly disagree) with statements on PDAs.* (N = 26)

**Quality of PDAs**	**Mean**	**SD**
Provide information at one's "fingertips"	2.00	0.75
A faster means to access information as compared to a *text reference *(e.g. CPS)	2.23	0.82
A faster means to access information as compared to a *desktop or laptop computer*	2.35	0.98
Help to organize information	2.46	0.90
Help to inform decisions in my patient care activities	2.50	0.99
An impetus to look up drug or disease information	2.65	1.20
Improve my patient's health outcomes	2.92	1.20
Decrease paperwork	3.35	1.16

* No significant differences were found between current personal digital assistant (PDA) and computer survey respondents' opinions (Fisher's Exact) regarding the PDA statements.

### Barriers and facilitators to personal digital assistants: themes from written comments

Peer support from colleagues, convenience, standardized usage, and financial and technical support were the main perceived facilitators to PDA use reported by respondents. The main perceived barrier to PDA use reported by respondents (n = 10) included cost. Other factors such as technology literacy, time, lack of peer support, no high speed internet for downloads, lack of needed resources, keeping up to date on resources, and searching speed were also reported.

### Future use of personal digital assistants

Fifty-two percent, including current PDA users, reported that they would use a PDA in the future. Twenty two percent were  uncertain and 19% reported that they would not use a PDA in the future. Two people did not respond.

### Confidentiality

Fifty two percent of respondents indicated that patient confidentiality with PDAs was no more concerning compared to use of other technologies. Forty-four percent did not know if they had a policy on patient confidentiality with regard to technologies.

### Technology training and reimbursement

Respondents rated (1 = least preferred to 5 = most preferred) one on one instruction and group learning led by an expert facilitator as the most preferred (mean 4.32, SD 0.99) means by which to receive instruction on a new technology. Least preferred methods included online discussions/chatrooms (mean 1.52, SD 1.04), internet videos (live: mean 1.70, SD 1.10, or static: mean 1.87, SD 1.14), video cassettes (mean 2.30, SD 1.55), trial and error learning (mean 2.32, SD 1.28), and written manuals (mean 2.92, SD 1.44). Paid leave for attendance at technology training sessions was the preferred means (mean 1.77, SD 0.86; 1 = strongly agree to 5 = strongly disagree) of remuneration for respondents. Respondents also indicated that if financial remuneration was to occur, it should correspond to the amount of time for training that is required (versus a flat rate) (mean 1.96, SD 1.08). Continuing education credits were not viewed as an incentive (mean 2.69, SD 1.44).

## Discussion

### Preferred resources

In our study, printed materials (e.g. compendia, journals, textbook resources) and professionals (e.g. pharmacists) were the most preferred and frequently used means to access information. Physician reliance on text and compendia relative to online/electronic resources has been frequently reported [11]. In a study examining family doctors' use of information sources to answer clinical questions, human resources (e.g. doctor, pharmacist), non-prescribing print information (e.g. textbooks and journal articles), and prescribing texts were used 36%, 32%, and 25% of the time, respectively [54]. Books from the workplace were reported by approximately 79% of UK primary care nurses as a commonly used source of knowledge and information used to support practice [55]. Fewer than one-third (31%) reported using electronic resources (e.g. Internet, electronic journals) for this purpose [55]. Results of a postal questionnaire to NPs demonstrated that 61% and 51% of respondents reported using drug reference manuals and textbooks, respectively, a few times a week or more [29]. These frequencies were second and third only to consulting with their physician supervisor (63%). Data from structured interviews of a sample of 22 community nurse prescribers reported by Hall et al. revealed that the majority relied on print materials to access information, namely the British National Formulary [32]. A survey of a primary care practice-based research network in the US that included physicians, physician assistants, and nurse practitioners, revealed that interpersonal and rapidly accessed print resources were preferred. Sixty-one and 58% of respondents reported using drug reference sources such as the Physician's Desk Reference (PDR) and medical textbooks, respectively, a few times a day or daily [56].

The clinicians in our sample perceived the Canadian compendium, the CPS, to be useful, accessible, credible, and current/timely. The CPS, is described as "the Canadian drug reference for health professionals" and is intended to provide a central source of drug information on drug products available in Canada [52]. It is available in print (English and French) and became available online in June 2004. The CPS includes drug monographs for commonly used products approved for use in Canada, but it does not include all drugs available on the Canadian market [57]. The majority of these product monographs are based on monographs submitted by pharmaceutical manufacturers and approved by Health Canada. Some of the monographs are written by the Canadian Pharmacists Association and are described as being evidence-based [52]. The CPS also includes more than 100 pages of clinical tools [52]. The CPS has been criticized for including pharmaceutical company advertising and requiring manufacturer payment for inclusion of product monographs [58]. The accuracy of particular components of CPS monographs has also been investigated. A review of overdose management in 119 monographs from the 2001 CPS revealed considerable variability in the utility of information with 50% of the monographs containing misleading or dangerous advice [59]. Since 2004, the CPS has included an alert box in the overdose section of monographs notifying users to contact Poison Control Centres for overdose management information. Some authors have criticized references that are similar to the CPS as being inadequate with regard to inclusion of evidence based information [60].

The NPs in our sample also rated Therapeutic Choices highly for all characteristics.  This finding is most likely  attributable to the fact that it is a recommended resource for coursework  associated with the Dalhousie NP university program curriculum.  Therapeutic Choices is a concise therapeutics reference text published by the Canadian Pharmacists Association. The text contains approximately 120 extensively referenced chapters with a disease management approach including easy to use algorithms and tables. An editorial board is responsible for extensively reviewing the content to ensure unbiased and objective information is presented [53].

### Health professionals

Reliance on other health professionals, especially pharmacists and physicians, as a resource for information was evident from our study and concurs with the findings of others [28,32,55,61]. Nurse practitioners have reported that collaborative relationships with pharmacists increase NP role satisfaction [61]. NPs frequently consult with allied health care professionals in their primary health care provider role and this is supported by written feedback from our sample regarding frequently consulted health professionals. Nursing colleagues are also likely to be rated highly by NPs due to their affiliation with peers from the same profession.

Some investigators have shown that non-human references (e.g. textbook) are sought for more technical aspects of prescribing (e.g. dose), whereas guidance regarding selection of agents (i.e. right drug for an indication) is sought from human resources (e.g. pharmacists or physicians) [62]. We were unable to determine what kinds of resources were used for specific purposes from our study.

### Online and electronic resources, computers, and personal digital assistants

From our study, computer survey respondents ranked online/electronic resources third in preference following print and health professionals. Various barriers and facilitators to accessing information online/electronically or via the Internet have been described in the literature [55,63-66]. Variables that have been described by others as barriers such as accessibility, high speed internet access, patient volume, age, practitioner type, and technology support did not appear to influence computer searching for information on drugs or therapeutics related to patient care in our results. Some qualitative feedback does however support this notion. As an example, in response to a request for a rationale for not using computers one physician commented: "Retro tech [sic]/old fashion. I still like to use my mind and have always been a fan of pen and paper". Barriers that were identified with our sample regarding the use of handheld technologies such as PDAs included cost, time, and issues related to technology literacy. Several people questioned the value of PDAs. One GP stated when referring to a PDA: "So far I have not discovered a use for one". Other respondents reinforced their preferences for other resources (e.g. books) and resistance to technology. When responding to barriers for the use of PDAs, one NP commented, "My huge dislike for machinery that frequently requires updating and patience". A physician responded, "as stated, I like to use my own mind, and can get all the info I need from books relatively quickly". Facilitators to the use of PDAs mainly included convenience factors such as having resources all in one place, faster means to get information, and portability. Our sample was not in agreement with some convenience factors in that they did not feel that PDAs would decrease paperwork. Practitioners from our sample felt relatively neutral about PDAs improving patient's health outcomes with 41% responding in this manner. Results from a sample of primary care practitioners in the US revealed that 76% agreed that the use of handheld devices for electronic prescribing would substantially reduce medical errors and improve the quality of health care [67].

Our study also suggests that resources such as the Cochrane Library and its Database of Systematic Reviews were not frequently used. This finding is similar to that of other investigators [30,35,64]. Despite the desire of some clinicians to use these resources, lack of confidence and ability to use them appropriately has been found [30,64,68,69]. Our study suggests that although this resource is perceived as credible, current/timely, and useful, it is also perceived to be somewhat inaccessible. The Cochrane Library is available to the health professionals (e.g. nurses, physicians, pharmacists, occupational therapists, physiotherapists, etc.) in our sample through professional bodies via the Atlantic Health Knowledge Partnership [70].

### Technology training: preferences and incentives

With regard to receiving training for a new technology, our study demonstrates that in person conferences or one on one training sessions are the preferred means to receive continuing education. Person to person interaction has been reported as the preferred and most frequently used means to access continuing education or training by other investigators [55,71].

Our study also indicates that this group of practitioners may benefit from accessing resources [72-80] that provide guidance on useful drug information resources available for devices such as PDAs. This is exemplified by one respondent's statement "knowledge regarding good software programs" as a barrier to the use of PDAs.

### Pharmaceutical industry

The influence of the pharmaceutical industry on physician prescribing and research outcomes has been documented [81,82]. Although NP use of industry representatives as a source of pharmacological information has been documented, the influence on prescribing is largely uninvestigated [32,61,83-85]. The CNA competency framework includes a statement regarding prescribing and industry relations [23]. In our study, the physician and NP rankings of industry representatives were similar. Within the health professionals and other category, pharmaceutical representatives were used as a resource by more of the sample than regional drug information services and comparably to academic detailing services. Academic detailing is a form of continuing  medical education where a trained health professional visits prescribers for  a fifteen to twenty minute session to provide objective information  regarding a therapeutic topic based on best available evidence [86,87]. Following academic detailing, physician and NP rankings of pharmaceutical industry representatives were second or third for frequency of use, usefulness, accessibility, credibility, and current/timeliness.

### Limitations

We do not have demographics or information regarding the reasons why survey recipients did not respond. As per ethical requirements to maintain confidentiality of respondents, we were not able to match respondents from their respective place of practice and therefore cannot conclude whether the practitioners within a practice setting influenced the others' responses. The sample size of the survey is small although it includes 88% response from community based NPs in Nova Scotia. The generalizability of the results is limited due to the variations in NP scopes of practice nationally and internationally. It is unknown whether the findings are generalizable to nonresponding physicians within Nova Scotia collaborating with NPs or to physicians not in collaborative practices with NPs as they were not included as a part of the sample. Due to multiple statistical comparisons (Mann Whitney U), the results comparing NP and physician ratings of results should be interpreted with caution.

## Conclusion

Respondent ratings of resources and preferences for resource use were consistent with self-reported means of conducting searches for specific drug information queries. The use of computers and PDAs remains limited and also matches preferences and resource ratings. Education to this group of practitioners regarding available drug information resources may facilitate use of computer and PDA resources. Further research is needed to determine methods to increase the use of computers and PDAs and if use of these technologies affects prescribing and patient outcomes.

## Competing interests

Ingrid Sketris holds a Chair from Canadian Institutes of Health Research (CIHR), Canadian Health Services Research Foundation (CHSRF) co-sponsored by the Nova Scotia Health Research Foundation (NSHRF). Andrea Murphy received salary support through this Chair as a research fellow at the time of conducting this research. The survey was performed in fulfillment of the requirements for the Drug Use Management and Policy Residency that Murphy participated in as a part of her fellowship. The residency was conducted with a decision making partner from the Nova Scotia Department of Health.

The opinions expressed in this paper are those of the authors and do not represent the opinions of the Nova Scotia Department of Health, CIHR/CHSRF or NSHRF.

MF, MM, RMM, and DG have no competing interests to declare.

## Authors' contributions

AM conceptualized the design and composed the survey instruments, carried out the study, entered and analyzed the data, drafted the original manuscript, and modified subsequent drafts based on authors' and reviewers' feedback. MF, RMM, IS, MM, and DG reviewed and suggested revisions to the survey tools, covering letters, overall study design, and contributed to feedback on the analysis and manuscript revisions.

## Pre-publication history

The pre-publication history for this paper can be accessed here:



## Supplementary Material

Additional file 1 **Computer survey**. Postal survey for computer users in PDF (Adobe Acrobat) format. Click here for file

Additional file 2 **PDA survey**. Postal survey for personal digital assistant (PDA) users in PDF (Adobe Acrobat) format. Click here for file

## References

[B1] Haynes RB, McKibbon KA, Fitzgerald D, Guyatt GH, Walker CJ, Sackett DL (1986). How to keep up with the medical literature I: why try to keep up and how to get started. Ann Intern Med.

[B2] Haynes RB, McKibbon KA, Fitzgerald D, Guyatt GH, Walker CJ, Sackett DL (1986). How to keep up with the medical literature II: deciding which journals to read regularly. Ann Intern Med.

[B3] Haynes RB, McKibbon KA, Fitzgerald D, Guyatt GH, Walker CJ, Sackett DL (1986). How to keep up with the medical literature III: expanding the number of journals you read regularly. Ann Intern Med.

[B4] Haynes RB, McKibbon KA, Fitzgerald D, Guyatt GH, Walker CJ, Sackett DL (1986). How to keep up with the medical literature IV: using the literature to solve clinical problems. Ann Intern Med.

[B5] Haynes RB, McKibbon KA, Fitzgerald D, Guyatt GH, Walker CJ, Sackett DL (1986). How to keep up with the medical literature V: access by personal computer to the medical literature. Ann Intern Med.

[B6] Haynes RB, McKibbon KA, Fitzgerald D, Guyatt GH, Walker CJ, Sackett DL (1986). How to keep up with the medical literature VI: how to store and retrieve articles worth keeping. Ann Intern Med.

[B7] Alper BS, Hand JA, Elliott SG, Kinkade S, Hauan MJ, Onion DK, Sklar BM (2004). How much effort is needed to keep up with the literature relevant for primary care?. J Med Libr Assoc.

[B8] Grol R, Grimshaw J (2003). From best evidence to best practice effective implementation of change in patients' care. Lancet.

[B9] (2005). The Cochrane Library. http://www3.interscience.wiley.com/cgi-bin/mrwhome/106568753/HOME.

[B10] Chassin MR (1998). Is health care ready for six sigma quality?. Milbank Q.

[B11] Westberg EE, Miller RA (1999). The basis for using the Internet to support the information needs of primary care. JAMIA.

[B12] Naylor CD (2002). Putting evidence into practice. Am J Med.

[B13] Graham ID, Beardall S, Carter AO, Glennie J, Hébert PC, Tetroe JM, McAlister FA, Visentin S, Anderson GM (2001). What is the quality of drug therapy clinical practice guidelines in Canada?. CMAJ.

[B14] Shaneyfelt TM, Mayo-Smith MF, Rothwangl J (1999). Are guidelines following guidelines? The methodological quality of clinical practice guidelines in the peer-reviewed medical literature. JAMA.

[B15] Johnston BL, Conly JM (2000). Guidelinitis: A new syndrome?. Can J Infect Dis.

[B16] Schwartz M Overview of the Canadian Federal Drug Review Process. http://www.hc-sc.gc.ca/ahc-asc/pubs/hpfb-dgpsa/overview-apercu_drug-med_rev_pro_03_07_e.html.

[B17] Rycroft-Malone J, Seers K, Titchen A, Harvey G, Kitson A, McCormack B (2004). What counts as evidence in evidence-based practice?. J Adv Nurs.

[B18] Banning M (2005). Conceptions of evidence, evidence-based medicine, evidence-based practice and their use in nursing: independent nurse prescribers' views. J Clin Nurs.

[B19] Sim I, Sanders GD, McDonald KM (2002). Evidence-based practice for mere mortals: the role of informatics and health services research. JGIM.

[B20] Tyler C, Hicks C (2001). The occupational profile and associated training needs of the nurse prescriber: an empirical study of family planning nurses. J Adv Nurs.

[B21] Maintaining Competency in Prescribing: An outline framework to help allied health professional supplementary prescribers. National Prescribing Centre, NHS. http://www.npc.co.uk/pdf/maintain_comp_in_presc_ofthahpsp.pdf.

[B22] College of Registered Nurses of Nova Scotia. Standards of Practice for Nurse Practitioners. http://www.crnns.ca/documents/CRNNS Standards of Practice Nurse Practitioners Sept 2005.pdf.

[B23] Canadian Nurse Practitioner Initiative Core Competency Framework. Canadian Nurses Association. http://www.cnpi.ca/documents/pdf/CNPE_Core_Competency_Framework_e.pdf.

[B24] Curran CR (2003). Informatics competencies for nurse practitioners. AACN Clinical Issues.

[B25] Otway C (2002). The development needs of nurse prescribers. Nurs Stand.

[B26] Latter S, Courtenay M (2004). Effectiveness of nurse prescribing: a review of the literature. J Clin Nurs.

[B27] Picton C, Granby T (2002). Maintaining and developing competencies in nurse prescribing. Br J Community Nurs.

[B28] Cogdill KW (2003). Information needs and information seeking in primary care: a study of nurse practitioners. J Med Libr Assoc.

[B29] Rasch R, Cogdill K (1999). Nurse practitioners' information needs and information seeking: implications for practice and education. Holist Nurs Pract.

[B30] Cullen RJ (2002). In search of evidence: family practitioners' use of the Internet for clinical information. J Med Libr Assoc.

[B31] Magrabi F, Coiera EW, Westbrook JI, Gosling AS, Vickland V (2005). General practitioners' use of online evidence during consultations. Int J Med Inform.

[B32] Hall J, Cantrill J, Noyce P (2003). The information sources used by community nurse prescribers. Br J Nurs.

[B33] Gosling AS, Westbrook JI, Spencer R (2002). Nurses' use of online clinical evidence. J Adv Nurs.

[B34] Gosling AS, Westbrook JI, Coiera EW (2003). Variation in the use of online clinical evidence: a qualitative analysis. Int J Med Inform.

[B35] Thompson C, McCaughan D, Cullum N, Sheldon TA, Mulhall A, Thompson DR (2001). Research information in nurses' clinical decision making: what is useful?. J Adv Nurs.

[B36] Government of Nova Scotia: Basic Facts about Nova Scotia. http://www.gov.ns.ca/cmns/overview/default.asp.

[B37] Rural Communities Impacting Policy Project 2003. Painting the Landscape of Rural Nova Scotia. Chapter 1: Demographics. http://www.ruralnovascotia.ca/RCIP/Demographics/Demographics.htm#PopulationOfCountyByAge.

[B38] Rural Communities Impacting Policy Project 2003 Painting the Landscape of Rural Nova Scotia. Chapter 5: Health. http://www.ruralnovascotia.ca/RCIP/Health/Health.htm.

[B39] Martin-Misener R, McNab J, Sketris IS, Edwards L (2004). Collaborative practice in health systems change: the Nova Scotia experience with the strengthening primary care initiative. Nurs Leadersh.

[B40] Nova Scotia Government Department of Health. Strengthening Primary Care Project Update July 24, 2002. http://www.gov.ns.ca/health/primaryhealthcare/projupdate.htm.

[B41] Nova Scotia Government Department of Health. Primary Health Care Reports: Strengthening Primacy Care Evaluation Results. http://www.gov.ns.ca/health/primaryhealthcare/reports.htm.

[B42] Graham L, Sketris IS, Burge F, Edwards L (2006). The effect of a primary care intervention on management of patients with diabetes and hypertension: a pre-post intervention chart audit. Healthc Q.

[B43] College of Registered Nurses of Nova Scotia. Nurse Practitioners Licensed in Nova Scotia. http://www.crnns.ca/default.asp?id=190&pagesize=1&sfield=content.id&search=1507&mn=414.70.81.412#primary.

[B44] College of Registered Nurses of Nova Scotia. Schedule of Drugs and Drug Interventions for Primary Health Care Nurse Practitioners in Nova Scotia. http://www.crnns.ca/default.asp?id=190&pagesize=1&sfield=content.id&search=1819&mn=414.70.81.412.

[B45] Anderson N, West M (1998). Measuring climate for work group innovation: development and validation of the team climate inventory. J Org Behav.

[B46] Gosling AS, Westbrook JI, Braithwaite J (2003). Clinical team functioning and IT innovation: a study of the diffusion of a point-of care online evidence system. J Am Med Inform Assoc.

[B47] Whelan AM, Nagpal S, Burge F (1994). Drug information resources in a family medicine residency training program. Can Pharm J.

[B48] Initiative for Medication Management, Policy Analysis, Research & Training, Dalhousie University. http://impart.pharmacy.dal.ca.

[B49] Dillman DA (1978). Mail and telephone surveys: the total design method.

[B50] Salant P, Dillman DA (1994). How to conduct your own survey.

[B51] Edwards P, Roberts I, Clarke M, DiGuiseppi C, Pratap S, Wentz R, Kwan I (2002). Increasing response rates to postal questionnaires: systematic review. BMJ.

[B52] Canadian Pharmacists Association (2005). Compendium of Pharmaceuticals and Specialties.

[B53] Gray J, Ed (2003). Therapeutics Choices.

[B54] Ely JW, Osheroff JA, Ebell MH, Bergus GR, Levy BT, Chambliss ML, Evans ER (1999). Analysis of questions asked by family doctors regarding patient care. BMJ.

[B55] Chan T, Brew S, de Lusignan S (2004). Community nursing needs more silver surfers: a questionnaire survey of primary care nurses' use of information technology. BMC Nursing.

[B56] Andrews JE, Pearce KA, Ireson C, Love MM (2005). Information-seeking behaviours of practitioners in a primary care practice-based research network (PBRN). J Med Libr Assoc.

[B57] Repchinsky C (2002). The CPS: love, hate and expectations. Can J Clin Pharmacol.

[B58] Bell RW, Osterman JW (1983). The Compendium of Pharmaceuticals and Specialties: a critical analysis. Int J Health Serv.

[B59] Brubacher JR, Purssell R, Kent DA (2002). Salty broth for salicylate poisoning? Adequacy of overdose management advice in the 2001 Compendium of Pharmaceuticals and Specialties. CMAJ.

[B60] Vidal L, Shavit M, Fraser A, Paul M, Leibovici L (2005). Systematic comparison of four sources of drug information regarding adjustment of dose for renal function. BMJ.

[B61] Lewis-Evans A, Jester R (2004). Nurse prescribers' experiences of prescribing. J Clin Nurs.

[B62] Sellman JS, Decarolis D, Schullo-Feulner A, Nelson DB, Filice GA (2004). Information resources used in antimicrobial prescribing. J Am Med Inform Assoc.

[B63] Darbyshire P (2003). "Rage against the machine?": nurses' and midwives experiences of using computerized patient information systems for clinical information. J Clin Inform.

[B64] Pritchard K, Chan T (2002). The confidence and competence of community nurses in using information and communications technology and in accessing clinical evidence through electronic libraries and databases. Inform Prim Care.

[B65] Dumas JA, Dietz EO, Connolly PM (2001). Nurse practitioner use of computer technologies in practice. Comput Nurs.

[B66] McAlearney AS, Schweikhart SB, Medow MA (2004). Doctors' experience with handheld computers in clinical practice: qualitative study. BMJ.

[B67] Andrews JE, Pearce KA, Sydney C, Ireson C, Love M (2004). Current state of information technology use in a US primary care practice-based research network. Inform Prim Care.

[B68] Haynes RB, McKibbon KA, Wilczynski NL, Walter SD, Werre SR (2005). Optimal search strategies for retrieving scientifically strong studies of treatment from Medline: analytical survey. BMJ.

[B69] Alpay L, Russell A (2002). Information technology training in primary care: the nurses' voice. Comput Inform Nurs.

[B70] Atlantic Health Knowledge Partnership. http://www.library.dal.ca/kellogg/ahkp/cochrane.htm.

[B71] Charles PA, Mamary EM (2002). New choices for continuing education: a statewide survey of the practices and preferences of nurse practitioners. J Cont Ed Nurs.

[B72] (2005). Recommendations for Handheld Hardware and Software. Dalhousie University Faculty of Medicine – Spring.

[B73] Drug Resources for Handheld PDA. RxFiles Academic Detailing. http://www.rxfiles.ca/acrobat/PDA-Drug-Resources-HEADER.pdf.

[B74] Miller SM, Beattie MM, Butt AA (2003). Personal digital assistant infectious diseases applications for health care professionals. Clin Infect Dis.

[B75] Keplar KE, Urbanski CJ (2003). Personal digital assistant applications for the healthcare provider. Ann Pharmacother.

[B76] Barrons R (2004). Evaluation of personal digital assistant software for drug interactions. Am J Health-Syst Pharm.

[B77] Clauson KA, Seamon MJ, Clauson AS, Van TB (2004). Evaluation of drug information databases for personal digital assistants. Am J Health-Syst Pharm.

[B78] McCreadie SR, Stevenson JG, Sweet BV, Kramer M (2002). Using personal digital assistants to access drug information. Am J Health-Syst Pharm.

[B79] Shneyder Y (2002). Personal digital assistants (PDA) for the nurse practitioner. J Pediatr Health Care.

[B80] Personal Digital Assistants - PDAs. College of Pharmacy, Drug Information.. http://pharmacy.dal.ca/druginfo/pda.html.

[B81] Watkins C, Moore L, Harvey I, Carthy P, Robinson E, Brawn R (2003). Characteristics of general practitioners who frequently see drug industry representatives: national cross sectional study. BMJ.

[B82] Lexchin J, Bero LA, Djulbegovic B, Clark O (2003). Pharmaceutical industry sponsorship and research outcome and quality: systematic review. BMJ.

[B83] Kessenich CR, Westbrook MH (1999). Pharmaceutical companies and the prescriptive practices of nurse practitioners. J Am Acad Nurse Pract.

[B84] Viale PH (2003). What nurse practitioners should know about direct-to-consumer advertising of prescription medications. J Am Acad Nurs Pract.

[B85] While AE, Biggs KSM (2004). Benefits and challenges of nurse prescribing. J Adv Nurs.

[B86] Faculty of Medicine, Continuing Medical Education, Dalhousie University: Academic Detailing Service. http://cme.medicine.dal.ca/ADS.htm.

[B87] Nova Scotia Health. Nova Scotia Establishes an Academic Detailing Service. http://www.gov.ns.ca/health/pharmacare/ads_summary.htm.

