# Supplementary material for: Drug information resources used by nurse practitioners and collaborating physicians at the point of care in Nova Scotia, Canada: a survey and review of the literature
Source: BMC Nurs. 2006 Jul 6;5:5. doi: 10.1186/1472-6955-5-5 (PMC1590010; doi:10.1186/1472-6955-5-5)
Supplement: Additional file 2 — PDA survey. Postal survey for personal digital assistant (PDA) users in PDF (Adobe Acrobat) format. [file 1472-6955-5-5-S2.pdf]

PDA Survey

If you *__use__* a PDA*
please complete *__this survey only__*.

*PDA = personal digital assistant
Examples include: Palm Pilot, Visor, Sony Clie

Please mark your answers in the boxes clearly with a ✓ or ✗.

Demographics

1. What is your current position?

   Physician ☐ Nurse practitioner ☐

   Other ☐ Please describe________________________

2. What is your age (in years)?

   ≤ 25 ☐ 46 – 55 ☐
   26 – 35 ☐ 56 – 65 ☐
   36 – 45 ☐ ≥ 66 ☐

3. What is your gender? Male ☐ Female ☐

4. How many patients on average do you see ***per day*** in a week?

   ≤ 15 ☐ 26 - 35 ☐ ≥ 46 ☐
   16 - 25 ☐ 36 - 45 ☐ ☐

5. Does your clinical practice have an Electronic Patient Record (EPR)?

   Yes ☐ No ☐

6. If yes, please estimate how long your clinical practice has had an EPR.

   < 6 months ☐ ≥ 1 year but < 3 years ☐
   ≥ 6 months but < 1 year ☐ ≥ 3 years ☐

PDA Use and Experience

7. Please indicate all that apply to describe your current PDA use in the following settings:

**Work**		**Yes**	**No**	**Home**		**Yes**	**No**
Used for searching drug/therapeutic information for patient care		☐	☐	Used for searching drug/therapeutic information for patient care		☐	☐
Hot syncing is performed here		☐	☐	Hot syncing is performed here		☐	☐
Length of use	< 1 year	☐		Length of use	< 1 year	☐	
	≥ 1 but < 5 years	☐			≥ 1 but < 5 years	☐	
	≥ 5 years	☐			≥ 5 years	☐	
Weekly use	>0 but < 5 hrs	☐		Weekly use	>0 but < 5 hrs	☐	
	≥ 5-10 hrs	☐			≥5-10 hrs	☐	
	≥11-15 hrs	☐			≥11-15 hrs	☐	
	>15 hrs	☐			>15 hrs	☐	

8. If you have access to a computer at work for hot sync operations, is this computer:

	Yes	No	Don't know
Shared	☐	☐	☐
Dedicated for your access	☐	☐	☐
High speed internet access equipped	☐	☐	☐

9. Does your workplace/employer:

	Yes	No	Don't know
provide PDA technical support?	☐	☐	☐
provide software or funds for PDA software?	☐	☐	☐

10. Please approximate the ***yearly personal cost*** for PDA updating/upkeep (this includes software purchases).

$ 0 ☐	$ ≥ 26 - 50 ☐	$ ≥ 76 - 100 ☐	$ ≥ 226 - 250 ☐
$ ≥ 1 - 25 ☐	$ ≥ 51 - 75 ☐	$ ≥ 101- 225 ☐	$ ≥ 251 ☐

11. Is the cost involved with maintaining your PDA:

Very reasonable ☐ **Reasonable** ☐ **Neutral** ☐ **Unreasonable** ☐ **Very unreasonable** ☐

In response to the **following statements regarding PDAs**, please indicate your level of agreement:

**"In my clinical practice, PDAs …**	**Strongly Agree**	**Agree**	**Neutral**	**Disagree**	**Strongly Disagree**
12. decrease paper work.	☐	☐	☐	☐	☐
13. help to organize information.	☐	☐	☐	☐	☐
14. provide information at one's "fingertips".	☐	☐	☐	☐	☐
15. are a faster means to access information as compared to a *desktop or laptop computer*.	☐	☐	☐	☐	☐
16. are a faster means to access information as compared to a *text reference* (e.g. CPS).	☐	☐	☐	☐	☐
17. help to inform decisions in my patient care activities.	☐	☐	☐	☐	☐
18. improve my patient's health outcomes.	☐	☐	☐	☐	☐
19. are an impetus to look up drug or disease information.	☐	☐	☐	☐	☐

20. Do you see yourself continuing to use a PDA in the future?

Yes ☐ No ☐ Don't know ☐

21. Please indicate the **frequency on a weekly basis** spent searching for the following drug information related issues in patient care:

Activity	Frequency			Activity	Frequency		
	Frequently	Infrequently	Never		Frequently	Infrequently	Never
Pediatric drug dosage	☐	☐	☐	Length of therapy	☐	☐	☐
Adult or usual drug dosage	☐	☐	☐	Drug use in pregnancy and/or lactation	☐	☐	☐
Geriatric drug dosage	☐	☐	☐	Toxicology/treatment of overdose or poisoning	☐	☐	☐
Dosage adjustment in organ dysfunction (e.g. renal)	☐	☐	☐	Monitoring (e.g. phenytoin levels; bloodwork frequency e.g. potassium)	☐	☐	☐
Indications	☐	☐	☐	Non-medicinal content of drugs (e.g. dyes)	☐	☐	☐
New indication(s) for older drugs	☐	☐	☐	Non-prescription/Over the counter drug information	☐	☐	☐
Information on new drugs	☐	☐	☐	Herbal therapy information	☐	☐	☐
Mechanism of action	☐	☐	☐	Identification of drugs	☐	☐	☐
Pharmacokinetics (e.g. half-life, metabolism, excretion),	☐	☐	☐	Formulary status (e.g. Nova Scotia Formulary)	☐	☐	☐
Dosage forms (e.g. liquid)	☐	☐	☐	Criteria for formulary exceptions status	☐	☐	☐
Side effects of drugs	☐	☐	☐	Cost of drugs	☐	☐	☐
Drug interactions (drug-drug, drug-food, drug-disease).	☐	☐	☐	Other<br>Please specify:			
Most appropriate drug for indication	☐	☐	☐				

22. Please choose **one** of the following to indicate **how (e.g. PDA, electronically via computer, or non-electronically/print)** searching is most often conducted for the items in question 21:

PDA only ☐ PDA > electronic > print ☐ Equal use of PDA, electronic & print ☐

Electronic only ☐ Electronic > PDA > print ☐

Print only ☐ Print > PDA or electronic ☐

23. Please indicate your **level of agreement** with the following statements for the ability of the **listed resources** to provide **drug and therapeutic information at the point of care in your practice setting:**

SA = Strongly Agree; A = Agree; N = neutral; DA = Disagree; SDA = Strongly Disagree; NA = Not applicable, I do not use the resource

**Resources:** ***Books, Journals, & Clinical Practice Guidelines***		**SA**	**A**	**N**	**DA**	**SDA**	**NA**
The **Compendium of Pharmaceuticals and Specialties** (CPS) is:	Used frequently	☐	☐	☐	☐	☐	☐
	Useful	☐	☐	☐	☐	☐	☐
	Accessible	☐	☐	☐	☐	☐	☐
	Credible	☐	☐	☐	☐	☐	☐
	Current and timely	☐	☐	☐	☐	☐	☐
**Therapeutic Choices** (published by Canadian Pharmacists' Association, edited by Jean Gray) is:	Used frequently	☐	☐	☐	☐	☐	☐
	Useful	☐	☐	☐	☐	☐	☐
	Accessible	☐	☐	☐	☐	☐	☐
	Credible	☐	☐	☐	☐	☐	☐
	Current and timely	☐	☐	☐	☐	☐	☐
**Specialty textbooks/handbooks** (e.g. Sanford Guide Antimicrobial Therapy) are:	Used frequently	☐	☐	☐	☐	☐	☐
	Useful	☐	☐	☐	☐	☐	☐
	Accessible	☐	☐	☐	☐	☐	☐
	Credible	☐	☐	☐	☐	☐	☐
	Current and timely	☐	☐	☐	☐	☐	☐
**Print journal subscriptions** are:	Used frequently	☐	☐	☐	☐	☐	☐
	Useful	☐	☐	☐	☐	☐	☐
	Accessible	☐	☐	☐	☐	☐	☐
	Credible	☐	☐	☐	☐	☐	☐
	Current and timely	☐	☐	☐	☐	☐	☐
**Print clinical practice guidelines** are:	Used frequently	☐	☐	☐	☐	☐	☐
	Useful	☐	☐	☐	☐	☐	☐
	Accessible	☐	☐	☐	☐	☐	☐
	Credible	☐	☐	☐	☐	☐	☐
	Current and timely	☐	☐	☐	☐	☐	☐

**Resources:** ***Online Resources***		**SA**	**A**	**N**	**DA**	**SDA**	**NA**
**Online journals** are:	Used frequently	☐	☐	☐	☐	☐	☐
	Useful	☐	☐	☐	☐	☐	☐
	Accessible	☐	☐	☐	☐	☐	☐
	Credible	☐	☐	☐	☐	☐	☐
	Current and timely	☐	☐	☐	☐	☐	☐
**Online bibliographic indexes** (e.g. Pubmed, CINAHL) are:	Used frequently	☐	☐	☐	☐	☐	☐
	Useful	☐	☐	☐	☐	☐	☐
	Accessible	☐	☐	☐	☐	☐	☐
	Credible	☐	☐	☐	☐	☐	☐
	Current and timely	☐	☐	☐	☐	☐	☐
**Online/electronic clinical practice guidelines** are:	Used frequently	☐	☐	☐	☐	☐	☐
	Useful	☐	☐	☐	☐	☐	☐
	Accessible	☐	☐	☐	☐	☐	☐
	Credible	☐	☐	☐	☐	☐	☐
	Current and timely	☐	☐	☐	☐	☐	☐
**The Cochrane Library** (www.cochrane.org) is:	Used frequently	☐	☐	☐	☐	☐	☐
	Useful	☐	☐	☐	☐	☐	☐
	Accessible	☐	☐	☐	☐	☐	☐
	Credible	☐	☐	☐	☐	☐	☐
	Current and timely	☐	☐	☐	☐	☐	☐
**Specialty and collection websites** (e.g. Medscape, theheart.org, RxFiles)	Used frequently	☐	☐	☐	☐	☐	☐
	Useful	☐	☐	☐	☐	☐	☐
	Accessible	☐	☐	☐	☐	☐	☐
	Credible	☐	☐	☐	☐	☐	☐
	Current and timely	☐	☐	☐	☐	☐	☐

Resources: *Professionals and Other*		SA	A	N	D	SDA	NA
**Physicians** are:	Used frequently	☐	☐	☐	☐	☐	☐
	Useful	☐	☐	☐	☐	☐	☐
	Accessible	☐	☐	☐	☐	☐	☐
	Credible	☐	☐	☐	☐	☐	☐
	Current and timely	☐	☐	☐	☐	☐	☐
**Nurse colleagues** are:	Used frequently	☐	☐	☐	☐	☐	☐
	Useful	☐	☐	☐	☐	☐	☐
	Accessible	☐	☐	☐	☐	☐	☐
	Credible	☐	☐	☐	☐	☐	☐
	Current and timely	☐	☐	☐	☐	☐	☐
**Pharmacists** are:	Used frequently	☐	☐	☐	☐	☐	☐
	Useful	☐	☐	☐	☐	☐	☐
	Accessible	☐	☐	☐	☐	☐	☐
	Credible	☐	☐	☐	☐	☐	☐
	Current and timely	☐	☐	☐	☐	☐	☐
**Other health professionals** (e.g. dieticians, occupational therapists, etc) are:	Used frequently	☐	☐	☐	☐	☐	☐
	Useful	☐	☐	☐	☐	☐	☐
	Accessible	☐	☐	☐	☐	☐	☐
	Credible	☐	☐	☐	☐	☐	☐
	Current and timely	☐	☐	☐	☐	☐	☐
**Online clinician discussion groups/email/listserves/ chatrooms** are:	Used frequently	☐	☐	☐	☐	☐	☐
	Useful	☐	☐	☐	☐	☐	☐
	Accessible	☐	☐	☐	☐	☐	☐
	Credible	☐	☐	☐	☐	☐	☐
	Current and timely	☐	☐	☐	☐	☐	☐
**Academic detailing services provided by Dalhousie Continuing Medical Education** are:	Used frequently	☐	☐	☐	☐	☐	☐
	Useful	☐	☐	☐	☐	☐	☐
	Accessible	☐	☐	☐	☐	☐	☐
	Credible	☐	☐	☐	☐	☐	☐
	Current and timely	☐	☐	☐	☐	☐	☐

Resources: *Professionals and Other*		SA	A	N	D	SDA	NA
**Regional drug information centres** (e.g. QE II Health Sciences Centre) are**:**	Used frequently	☐	☐	☐	☐	☐	☐
	Useful	☐	☐	☐	☐	☐	☐
	Accessible	☐	☐	☐	☐	☐	☐
	Credible	☐	☐	☐	☐	☐	☐
	Current and timely	☐	☐	☐	☐	☐	☐
**Pharmaceutical industry medical information centers** are**:**	Used frequently	☐	☐	☐	☐	☐	☐
	Useful	☐	☐	☐	☐	☐	☐
	Accessible	☐	☐	☐	☐	☐	☐
	Credible	☐	☐	☐	☐	☐	☐
	Current and timely	☐	☐	☐	☐	☐	☐
**Pharmaceutical industry representatives** are:	Used frequently	☐	☐	☐	☐	☐	☐
	Useful	☐	☐	☐	☐	☐	☐
	Accessible	☐	☐	☐	☐	☐	☐
	Credible	☐	☐	☐	☐	☐	☐
	Current and timely	☐	☐	☐	☐	☐	☐
**Other**: please describe:	Used frequently	☐	☐	☐	☐	☐	☐
	Useful	☐	☐	☐	☐	☐	☐
	Accessible	☐	☐	☐	☐	☐	☐
	Credible	☐	☐	☐	☐	☐	☐
	Current and timely	☐	☐	☐	☐	☐	☐

SA = Strongly Agree; A = Agree; N = neutral;
DA = Disagree; SDA = Strongly Disagree;
NA = Not applicable, I do not use the resource

Preferred Drug/Therapeutic Information Resource

24. For the broad categories of references in **question 23** and as listed below, please rate them from least to most preferred if you could choose from all means of accessing drug or therapeutic information at the point of care (i.e. when seeing patients/clients): (**1 = least preferred to 5 = most preferred**).

	1	2	3	4	5
PDA based resources	☐	☐	☐	☐	☐
Books, Journals, & Print Clinical Practice Guidelines	☐	☐	☐	☐	☐
Online resources	☐	☐	☐	☐	☐
Other health professionals	☐	☐	☐	☐	☐

Please indicate health professional(s) with whom you would consult (e.g. physiotherapist, dietician, physician, nurse practitioner, pharmacist, etc):

____

____

25. Please indicate the names of software programs or sources that you currently use or have access to for your PDA:

Category	Name of program or resource	Do not have
Drug references		☐
Patient education information		☐
Clinical Calculators		☐
Patient trackers		☐

26. If you have used other drug reference software program(s) or other sources that differ from your current program(s) from **question 25** please indicate the name(s) of these and the reason(s) for which you no longer use them:

Program or resource	Reason for discontinuation of use

27. Please indicate the features and/or programs that you would like to have access to on your PDA:

Feature or program	Yes	No	Does not matter
Ability to track clinical activities/statistics	☐	☐	☐
Nova Scotia formulary (Pharmacare)	☐	☐	☐
Nova Scotia formulary exception status forms	☐	☐	☐
Clinical practice guidelines (Canadian)	☐	☐	☐
Clinical practice guidelines (other countries)	☐	☐	☐
Clinical calculators (e.g. body mass index)	☐	☐	☐
Drug monographs	☐	☐	☐
Patient education information	☐	☐	☐
Other (please describe)			

PDA barriers, facilitators, and confidentiality:

28. What **barriers** do you see for the use of PDAs in your practice setting?

29. What **facilitators** do you see for the use of PDAs in your practice setting?

30. Are you concerned about **patient confidentiality** for information entered in PDAs:

Yes ☐ **No more than with other means of tracking patient information** ☐

No ☐

Other: **please describe**

31. Does your practice setting currently have a **policy on patient confidentiality** in relation to electronic technology such as electronic patient records or PDAs?

Yes ☐ No ☐ Don't know ☐

If yes, please briefly describe your policy.

32. If you answered no to **question 31**, are there plans to develop or adapt a policy in your practice?

Yes ☐ No ☐ Don't know ☐

Technology training:

33. If you were in need of **training** regarding a new electronic tool or software program used for clinical decision-making please **rank** the following selections according to your preferences for receiving training. (**1 = least preferred to 5 = most preferred**).

	1	2	3	4	5
One on one instruction	☐	☐	☐	☐	☐
Group learning lead by an expert facilitator	☐	☐	☐	☐	☐
A written instruction manual	☐	☐	☐	☐	☐
Independent learning with trial and error	☐	☐	☐	☐	☐
An internet chat group	☐	☐	☐	☐	☐
An online video on the internet that can be played repeatedly	☐	☐	☐	☐	☐
A live video that can be viewed on the internet	☐	☐	☐	☐	☐
A video cassette (VHS) or DVD	☐	☐	☐	☐	☐
Other? Please describe	☐	☐	☐	☐	☐

	Please indicate your **level of agreement** with the following statements:	Strongly Agree	Agree	Neutral	Disagree	Strongly Disagree
34.	I would be *more likely* to attend a training program as indicated in question 33 if **continuing education credits** were offered.	☐	☐	☐	☐	☐
35.	I would be *more likely* to attend a training program as indicated in question 33 if **financial remuneration** was offered.	☐	☐	☐	☐	☐
36.	I would be *more likely* to attend a training program as indicated in question 33 if **paid leave** was offered.	☐	☐	☐	☐	☐
37.	I would be *more likely* to attend a training program as indicated in question 33 **if the remuneration** (financial, time off, continuing education credits) **corresponded to the amount of time required for training** versus a flat rate for the entire training.	☐	☐	☐	☐	☐

Thank you again for taking the time to complete this survey.
